# Acute-phase reactants after paediatric cardiac arrest. Procalcitonin as marker of immediate outcome

**DOI:** 10.1186/1471-2431-8-18

**Published:** 2008-04-30

**Authors:** Marta Los Arcos, Corsino Rey, Andrés Concha, Alberto Medina, Belen Prieto

**Affiliations:** 1 Paediatric Intensive Care Unit, Department of Paediatrics, Hospital Universitario Central de Asturias, University of Oviedo, Oviedo, Spain; 2 Clinical Biochemistry Laboratory. Hospital Universitario Central de Asturias, University of Oviedo, Oviedo, Spain

## Abstract

**Objective:**

Procalcitonin (PCT) and C reactive protein (CRP) have been used as infection parameters. PCT increase correlates with the infection's severity, course, and mortality. Post-cardiocirculatory arrest syndrome may be related to an early systemic inflammatory response, and may possibly be associated with an endotoxin tolerance. Our objective was to report the time profile of PCT and CRP levels after paediatric cardiac arrest and to assess if they could be use as markers of immediate survival.

**Materials and methods:**

A retrospective observational study set in an eight-bed PICU of a university hospital was performed during a period of two years. Eleven children younger than 14 years were admitted in the PICU after a cardiac arrest. PCT and CRP plasma concentrations were measured within the first 12 and 24 hours of admission.

**Results:**

In survivors, PCT values increased 12 hours after cardiac arrest without further increase between 12 and 24 hours. In non survivors, PCT values increased 12 hours after cardiac arrest with further increase between 12 and 24 hours. Median PCT values (range) at 24 hours after cardiac arrest were 22.7 ng/mL (0.2 – 41.0) in survivors vs. 205.5 ng/mL (116.6 – 600.0) in non survivors (p < 0.05). CRP levels were elevated in all patients, survivors and non-survivors, at 12 and 24 hours without differences between both groups.

**Conclusion:**

Measurement of PCT during the first 24 hours after paediatric cardiac arrest could serve as marker of mortality.

## Background

Procalcitonin (PCT) and C reactive protein (CRP) have been used as infection parameters. PCT concentration increases because of several microbial infections and this increase correlates with the infection's severity, course, and mortality [1-5]. Other studies have shown low to only moderately elevated PCT in uninfected patients after major trauma, surgery and cardiogenic shock [6-8]. However, median values under these conditions are usually below those found in severe sepsis and septic shock. Several studies have reported higher PCT levels in non-survivors, compared with survivors, after severe sepsis, cardiac surgery or trauma patients [9-12].

Paediatric cardiac arrest is associated with high in-hospital mortality [13]. Only one-third of children who suffer a cardiac arrest when admitted to a Pediatric Intensive Care Unit (PICU) survive. The duration of cardiopulmonary resuscitation (CPR) attempts and the doses of epinephrine are the best indicators of mortality[14]. Adrie C et al [15] found a marked increase in plasma cytokines of patients successfully resuscitated after cardiac arrest, especially in nonsurvivors. Ischemic reperfusion injury is closely related to neutrophils activated by cytokines. Therefore, it has been hypothesized that post-cardiocirculatory arrest syndrome may be related to an early systemic inflammatory response, leading to an exacerbation of the inflammatory balance, and may possibly be associated with an endotoxin tolerance. This condition has many features in common with sepsis. Several biochemical variables, including neuron specific enolase and interleukin-8, have been described as biochemical markers of cerebral damage [16].

A recent study has shown that PCT serum levels were significantly higher in cardiac arrest patients who died of refractory shock than in those who died of neurological failure or survive[17]. There are not references in the literature about the correlation of both, PCT and CRP, and mortality after cardiac arrest in children. Our objective was to report the time profile of PCT and CRP levels after paediatric cardiac arrest and to assess if they could be use as markers of immediate survival.

## Methods

A retrospective observational study set in an eight-bed PICU of a university hospital was performed. During a period of two years, eleven children younger than 14 years were admitted in our PICU after a cardiac arrest. PCT and CRP plasma concentrations were measured within the first 12 and 24 hours of admission. Plasma CRP was measured on a Vitros 5.1 Fusion Chemistry System (Ortho Clinical Diagnostics, Buckinghamshire, UK). For the determination of PCT, time-resolved amplified cryptate emission (TRACE) technology was used on a Kryptor analyzer (Brahms, Hennigsdorf, Germany). Limits of detection were 0.3 mg/dl and 0.02 ng/ml for CRP and PCT methods, respectively. The study received ethics approval by the Hospital Ethics Research Committee.

We compared CRP and PCT plasma levels among survivor and non survivor patients. Mann-Whitney *U*-test was used to compare two independent samples. A *p*-value ≤ 0.05 was considered significant.

## Results

Eleven children, six males successfully resuscitated after cardiac arrest were admitted in our PICU. Seven patients survived and four did not survive at hospital discharge. Age, diagnosis, place of arrest, cardiac arrest duration, outcome and cause of death for each individual patient are shown in Table 1. Children who died suffered out-hospital cardiac arrest of longer duration than children who survive.

**Table 1:** Patient characteristics, place of arrest, cardiac arrest duration, outcome and cause of death.

Case	Age (months)	Diagnosis	Place of arrest	Estimated time to initiation of CPR (minutes)	Time of CRA (minutes)	Outcome (hospital discharge)	Cause of death
1	162	Drowning	Out-hospital	15	35	Death	Brain death
2	42	Drowning	Out-hospital	20	31	Death	Withdrawal of care
3	5	Sudden death	Out-hospital	10	30	Death	Brain death
4	151	Drowning	Out-hospital	15	50	Death	Brain death
5	178	Ventricular arrytmia	Out-hospital	1	7	Normal neurological function	Alive
6	8	Obstructive hydrocefalia	In-hospital	0	3	Normal neurological function	Alive
7	6	Bronchiolitis	In-hospital	0	4	Normal neurological function	Alive
8	2	Bronchiolitis	In-hospital	0	3	Normal neurological function	Alive
9	21	Drowning	Out-hospital	10	11	Neurological damage	Alive
10	21	Pneumonia	In-hospital	0	1	Normal neurological function	Alive
11	4	Heat stroke Hyperkaliemia	In-hospital	0	8	Normal neurological function	Alive

CPR: cardiopulmonar resuscitation

CRA: cardiac arrest

Median PCT values (range) at 12 hours after cardiac arrest were 23.6 ng/mL (3.1 – 78.2) and 79.1 ng/mL (32.8 – 300.8) in survivors and non survivors, respectively (p NS), whereas at 24 hours were 22.7 ng/mL (0.2 – 41.0) and 205.5 ng/mL (116.6 – 600.0) in survivors and non survivors, respectively (p < 0.05). Median CRP values at 12 hours were 2.1 mg/dL (0.4 – 8.5) and 1.3 mg/dL (0.1 – 3.0) in survivors and non survivors, respectively (p NS) whereas at 24 hours were 9.1 mg/dL (2.0 – 24.4) and 4.5 mg/dL (4.0 – 10.5) in survivors and non survivors, respectively (p NS).

**Figure 1:**
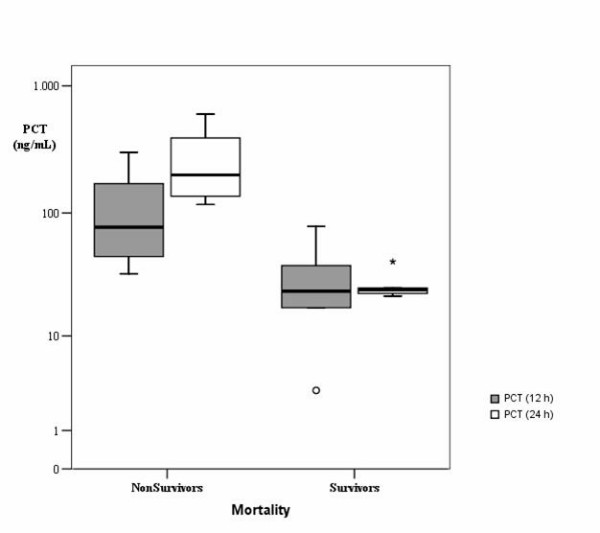
**Box-plot of PCT plasma concentrations in the different groups.** Data are presented in a logarithmic scale. *Central line*: median; *boxes*: 25th to 75th percentiles; *whiskers*: 95% confidence. P < 0.05 for PCT values at 24 hours between survivors and non survivors.

**Figure 2:**
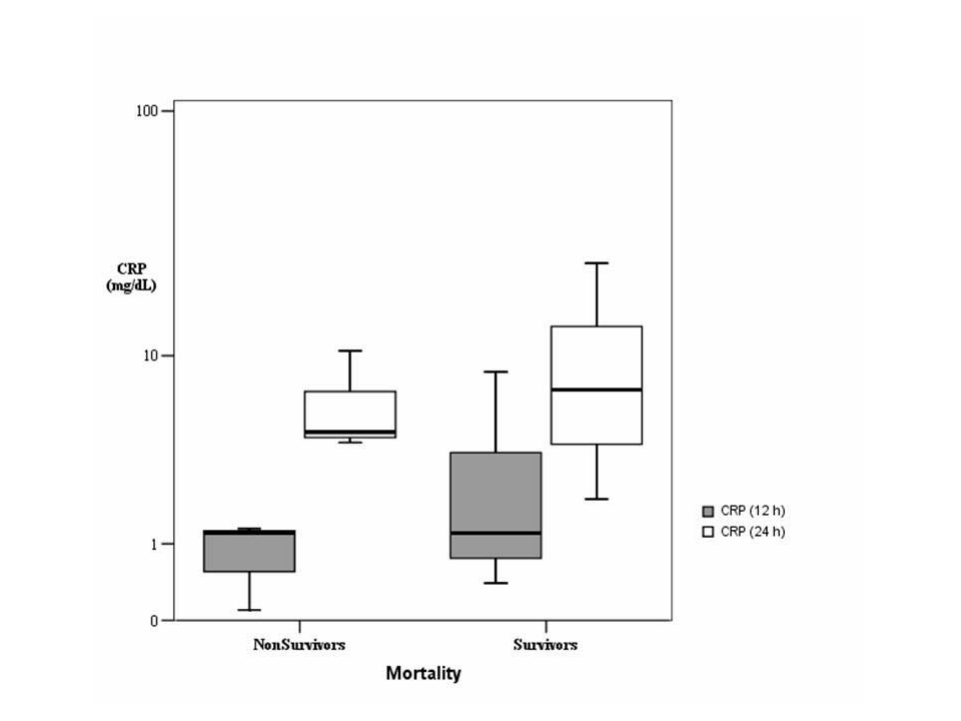
**Box-plot of CRP plasma concentrations in the different groups.** Data are presented in a logarithmic scale *Central line*: median; *boxes*: 25th to 75th percentiles; *whiskers*: 95% confidence.

Figures 1 and 2 show box-plots of PCT and CRP plasma concentrations in survivor and no survivor groups. In survivors, PCT values increased 12 hours after cardiac arrest, without further increase between 12 and 24 hours. In non survivors, PCT values increased 12 hours after cardiac arrest with further increase between 12 and 24 hours. Twenty-four hours after admission, PCT values were higher than 100 ng/mL in non survivor patients, and lower than 41 ng/mL in survivor patients (p < 0.05). CRP levels were elevated in all patients, survivors and non-survivors 12 and 24 hours after admission without differences between both groups.

**Figure 3:**
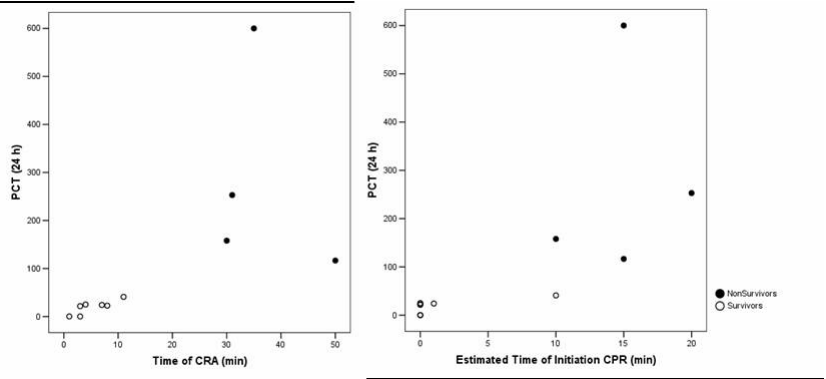
Correlation between time of cardiac arrest (CRA) and time to initiation of cardiopulmonary resuscitation (CPR) with procalcitonin (PCT) values at 24 h.

**Figure 4:**
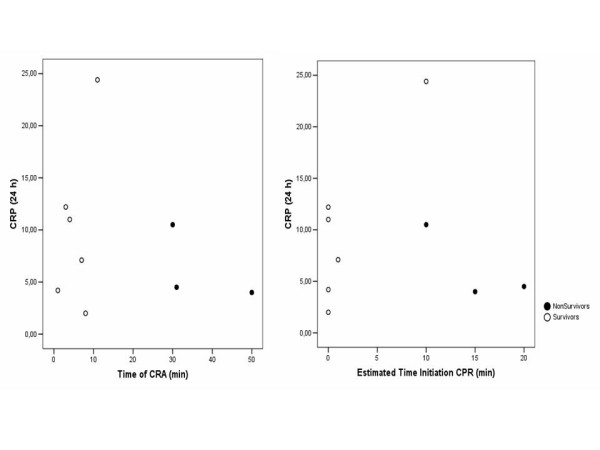
Correlation between time of cardiac arrest (CRA) and time to initiation of cardiopulmonary resuscitation (CPR) with C reactive protein (CRP) values at 24 h.

In spite of the reduced number of patients, it is interesting to point out that time of cardiac arrest and time to initiation of CPR were correlated with PCT values at 24 h (Figure 3). This relation was not found with 24 h PCR, since similar PCR levels were observed regardless of these times (Figure 4).

## Discussion

We found in a small group of paediatric patients that PCT levels higher than 100 ng/mL 24 hours after cardiac arrest may identify high risk of mortality. Several studies have demonstrated PCT utility for the diagnosis of severe bacterial infections[1-5,18]. Its plasma levels have also been related with severity of sepsis[1,4,5,10,11,19]. Serial PCT measurements are useful as a prognosis marker. A fall in PCT after 24 hours of treatment may have favorable prognostic significance [19]. On the other hand, increasing or high PCT levels may indicate a poor prognosis. In septic and non septic patients, PCT levels were found to be highly predictive for mortality and organ failure development[1,9,11,19]. During cardiac arrest delivery of oxygen is abruptly halted. After recovery of spontaneous circulation, ischemia/reperfusion syndrome causes an early systemic inflammatory response, leading to an exacerbation of the inflammatory balance, and may possibly be associated with an endotoxin tolerance. This condition has many features in common with sepsis. Plasma levels of various cytokines, soluble receptors, and endotoxin were associated with outcome after cardiac arrest [15]. PCT is produced in the liver and other tissues following activation by mononuclear cells[20]. A PCT production is also reported in shock situations[2,8,19,21], after surgery and tissue trauma[22]. Cardiac arrest is the most severe shock situation in a critical child. In all our children, survivors and non-survivors, PCT and CRP levels increased at 12 and 24 hours after cardiac arrest, confirming that both markers are elevated in patients with shock. PCT increase has been described after cardiac arrest in adults[23,24]. Comparing both groups, non-survivors maintained PCT increase between 12 and 24 hours whereas survivors did not increase PCT levels. This could indicate that non survivor patient's present a more severe shock with more severe tissue damage, hypoxemia and reperfusion injury, associated with longer time of cardiac arrest, mortality and PCT increase. In our patients, time of cardiac arrest and time to initiation of CPR were correlated with PCT values at 24 h. We did not find this quality for CRP.

Another important finding is that PCT elevation is not directly related with infection in these cases. According with the literature, cut-off PCT values for diagnosis of bacterial infections are between 0.5 and 5.0 ng/mL whereas cut-off values for diagnosis of shock are much higher[1,5,18]. Splanchnic hypoperfursion after cardiac arrest as well as reperfusion injury can cause mucosal barrier damage permitting translocation of endotoxin to the bloodstream and increasing PCT to the high values detected in our patients.

The main limitation of our study is the small number of patients. Because cardiac arrest is uncommon in paediatric age a multicenter study could be necessary to have a bigger sample.

## Conclusion

Measurement of PCT during the first 24 hours after paediatric cardiac arrest can predict non survival of patients. PCT values higher than 100 ng/mL 24 hours after cardiac arrest could serve as marker of mortality. CRP did not show this quality.

## Abbreviations

PCT: procalcitonin; CRP: C-reactive protein; CRA: cardiac arrest; PICU: paediatric intensive care unit; CPR: cardiopulmonar resuscitation.

## Competing interests

CR has received remuneration for holding lectures on the topic of prognosis markers of sepsis by BRAHMS, Germany. The authors declare that they have no further competing interests.

## Authors' contributions

MLA participated in the design of the study, analyzed data and drafted the manuscript. CR conceived the study, participated in the design and drafted the manuscript. AC and AM participated in the design, and reviewed the manuscript. BP was responsible for biochemical determinations and reviewed the manuscript. All authors read and approved the final manuscript.

## Pre-publication history

The pre-publication history for this paper can be accessed here:


